# Metallic Phase Transition Metal Dichalcogenide Quantum Dots as Promising Bio-Imaging Materials

**DOI:** 10.3390/nano12101645

**Published:** 2022-05-11

**Authors:** Kwang Hyun Park, Jun Yong Yang, Sunggyeong Jung, Byoung Min Ko, Gian Song, Soon-Jik Hong, Nam Chul Kim, Dongju Lee, Sung Ho Song

**Affiliations:** 1 Division of Advanced Materials Engineering, Center for Advanced Powder Materials and Parts, Kongju National University, Cheonan 32588, Chungnam, Korea; recite14@gmail.com (K.H.P.); yajy2306@naver.com (J.Y.Y.); jsk71317@gmail.com (S.J.); qudals3920@gmail.com (B.M.K.); gasong@kongju.ac.kr (G.S.); hongsj@kongju.ac.kr (S.-J.H.); 2 Department of Advanced Materials Engineering, Chungbuk National University, Chungdae-ro 1, Seowon-gu, Cheongju 34057, Chungbuk, Korea

**Keywords:** quantum dots, transition metal dichalcogenide, bio-imaging, density functional theory, quantum confinement

## Abstract

Transition metal dichalcogenide-based quantum dots are promising materials for applications in diverse fields, such as sensors, electronics, catalysis, and biomedicine, because of their outstanding physicochemical properties. In this study, we propose bio-imaging characteristics through utilizing water-soluble MoS_2_ quantum dots (MoS_2_-QDs) with two different sizes (i.e., ~5 and ~10 nm). The structural and optical properties of the fabricated metallic phase MoS_2_-QDs (*m*-MoS_2_-QDs) were characterized by transmission electron microscopy, atomic force microscopy, X-ray photoelectron spectroscopy, Raman spectroscopy, UV–vis absorption spectroscopy, and photoluminescence. The synthesized *m*-MoS_2_-QDs showed clear photophysical characteristic peaks derived from the quantum confinement effect and defect sites, such as oxygen functional groups. When the diameter of the synthesized *m*-MoS_2_-QD was decreased, the emission peak was blue-shifted from 436 to 486 nm under excitation by a He-Cd laser (325 nm). Density functional theory calculations confirmed that the size decrease of *m*-MoS_2_-QDs led to an increase in the bandgap because of quantum confinement effects. In addition, when incorporated into the bio-imaging of HeLa cells, *m*-MoS_2_-QDs were quite biocompatible with bright luminescence and exhibited low toxicity. Our results are commercially applicable for achieving high-performance bio-imaging probes.

## 1. Introduction

Transition metal dichalcogenides (MX_2_, where M = Mo, W and X = S, Se), which consist of S-Mo-S triple layers bound by weak van der Waals forces, have attracted great attention owing to their high electrochemical activity, carrier transport efficiency, excellent light–heat conversion, and fluorescence imaging properties [1,2,3,4,5,6]. The MX_2_ layer compounds show unique electric and optical properties ascribed to polymorphic phase structures, such as metallic (1T/1T′) and semiconducting (2H) phases [4,7]. In addition, the electric and optical properties of the MX_2_ layer compounds have been tailored to the quantum confinement effect, such as MX_2_ quantum dots (QDs) and surface states through doping and chalcogen defects in crystals [8,9]. Among them, MX_2_-QDs have led to significant improvements in bio-sensing and bio-imaging owing to their good dispersibility, strong fluorescence emission, and low toxicity, compared to traditional semiconductor materials, such as CdS and CdSe [10,11]. However, a theoretical understanding of the optical properties and mass production of MX_2_-QDs is required for realizing practical applications in bio-imaging fields.

As a typical MX_2_, MoS_2_ shows a transition from an indirect to a direct bandgap due to quantum confinement with decreasing layer thickness. In addition, MoS_2_ quantum dots (MoS_2_-QDs) exhibit a high quantum yield and a broadening of excitonic absorption owing to the enhanced spin–valley coupling and strong exciton binding energy [12]. Furthermore, recent studies about semiconductor QDs (MX_2_-based QDs) successfully suggested their potential in memory devices and neuromorphic computing because of the tunable electrical/optical characteristics of the QDs [13]. On the other hand, research on metallic-MoS_2_ QDs (*m*-MoS_2_-QDs), which possess interesting characteristics, such as photophysical properties, is rarely reported because of the metastable nature of the metallic phase compared with that of semiconducting MoS_2_-QDs (e.g., 2H-MoS_2_-QDs).

To date, the fabrication routes of MoS_2_-QDs can be generally classified as bottom-up or top-down approaches. Ren et al. synthesized MoS_2_ QDs using dibenzyl disulfides and sodium molybdate as sulfur and molybdenum sources, respectively, via a bottom-up method. Top-down approaches for MoS_2_-QDs fabrication using various methods, including the intercalation of alkali metals, solvothermal treatment, photo-Fenton reaction, and liquid exfoliation [13,14], have been suggested. Xu et al. demonstrated the synthesis of MoS_2_- and WS_2_-QDs through solvothermal treatment [15]. Xu et al. and Li et al. reported a size-controllable method for highly luminescent MoS_2_-QDs based on the photo-Fenton reaction [16,17]. Metallic 1T-MoS_2_-QDs were prepared via hydrothermal synthesis and lithium intercalation of bulk MoS_2_ [18,19]. However, these approaches are limited by various operating conditions, such as long fabrication times, low yields, and the use of toxic reagents.

In this study, we propose a simple, eco-friendly, and scalable route for fabricating *m*-MoS_2_-QDs with different sizes (*m*-MoS_2_-QD I: ~5 nm, *m*-MoS_2_-QD II: ~10 nm) via the co-intercalation of potassium sodium tartrate (KNaC_4_H_4_O_6_·4H_2_O) and exfoliation in water, without the use of toxic chemical solvents or surfactants. The structural and optical properties of the *m*-MoS_2_-QDs were assessed by transmission electron microscopy (TEM), atomic force microscopy (AFM), X-ray photoelectron spectroscopy (XPS), Raman spectroscopy, UV–vis absorption spectroscopy, and photoluminescence (PL). In particular, the optical properties of *m*-MoS_2_-QDs are affected by the quantum confinement effect and defects, which were assessed by comparison of the results of density functional theory (DFT) calculations and structural analysis. In addition, the biocompatibility and toxicity of the *m*-MoS_2_-QDs were monitored.

## 2. Materials and Methods

### 2.1. Preparation of m-MoS_2_-QDs

The *m*-MoS_2_-QDs were fabricated using potassium sodium tartrate (Sigma-Aldrich, St. Louis, MO, USA) with Na and K metal ions in MoS_2_ bulk powder at low temperature. After MoS_2_ powder (200 mg) and potassium sodium tartrate (20 mg) were mixed by grinding, the compound was kept at 250 °C for 12 h in an autoclave chamber. Then, the resulting compound was dispersed in distilled water and exfoliated to MoS_2_ QDs by sonication for 1 h in a water bath. After a purification process using a 10 KDMWCO dialysis tube for 24 h, the size-sorting of synthesized *m*-MoS_2_-QDs with a wide range of sizes was carried out via centrifuge filtration process (i.e., 8000 and 10,000 NMWL, Amicon Ultra-15). The centrifugation process was conducted at 5000 rpm for 30 min. Finally, *m*-MoS_2_–QD I and m-MoS_2_–QD II powders were obtained after a freeze-drying process [12,20].

### 2.2. Characterization

Morphology analyses of *m*-MoS_2_-QDs were conducted using AFM (SPA400, SII, Chiba, Japan) under ambient conditions in the tapping mode. High-resolution transmission electron microscopy (HR-TEM, Tecnai G2 F30, Hillsboro, OR, USA) was measured after the droplet of the QD suspension was placed on the TEM grid. Raman spectroscopy (LabRAM HR UV/Vis/NIR, excitation at 514 nm, Horiba, Palaiseau, France) was used for the measurement of structural properties of *m*-MoS_2_-QDs. The chemical properties of the *m*-MoS_2_-QDs were measured by XPS (Sigma Probe, AlKα, Thermo Fisher Scientific, Kyoto, Japan) and FT-IR (FT-IR-4100 type-A, JASCO, Portland, OR, USA) spectra. The optical properties of the *m*-MoS_2_-QDs were analyzed by UV–vis spectroscopy (Maya2000, Ocean Optics, Dunedin, FL, USA) and fluorescence spectra (Perkin-Elmer LS 55 luminescence spectrometer, Perkin-Elmer Ltd., Llantrisant, UK) at room temperature. PL measurements were obtained using a 325 nm He-Cd continuous-wave laser with monochromatic light from a 300 W xenon lamp (Coherent, Chameleon Ultra II, Santa Clara, CA, USA).

### 2.3. Density Functional Calculations

DFT calculations were conducted using the generalized gradient approximation for the exchange-correlation potential [21] and projector-augmented wave potentials [22], as implemented in the VASP code [23]. The wave functions were expanded in plane waves up to an energy cutoff of 400 eV. We employed a supercell geometry with a vacuum region of more than 8 Å to prevent interactions between adjacent supercells.

### 2.4. Cytotoxicity Evaluation

The cells were cultured and maintained in DMEM low glucose medium (WelGene Inc., Daegu, Korea) comprising 1 g/mL D-glucose, 4 mM L-glutamine, and 110 mg mL^−1^ sodium pyruvate, with 10% fetal bovine serum, 100 IU mL^−1^ penicillin, and 100 µg mL^−1^ streptomycin. Freshly isolated mouse primary hepatocytes were plated at 3 × 105 cells per well in 6-well plates and left to adhere overnight. Various concentrations (0–100 µg mL^−1^) of *m*-MoS_2_-QDs in the culture media were added to each well in duplicate. The cells were incubated for 24 h. Finally, cell soups were collected, and LDH level tests were carried out using a VetTest Chemistry analyzer. The experimental method is similar to previous work [24].

### 2.5. Cell Imaging

Cells were plated in a 6-well plate at a density of 3 × 105 cells per well, and each well contained 2 mL of culture medium. After overnight culture, 50 µg mL^−1^ of *m*-MoS_2_-QDs was added to the culture medium and incubated in regular cell culture conditions for 24 h. Then, the medium with *m*-MoS_2_-QDs was removed from the cells and washed twice with 1 mL 1X filtered PBS. Cells were fixed using 1% paraformaldehyde and mounted with Vectashield antifade mounting medium with DAPI (Vector Laboratories, Inc., Burlingame, CA, USA). For concentration-dependent cell imaging, cytotoxicity test samples were used. Cellular imaging was performed using a LEICA DMI 4000 B microscope equipped with a DFC450C camera (Leica, Germany). A LEICA CTR 4000 lamp (Leica, Germany) was used as the fluorescence light source. For multiphoton imaging at 790 nm, a Zeiss LSM 510 META confocal microscope (Carl Zeiss, Jena, Germany) was used. Experimental steps are similar to previous work [24].

## 3. Results and Discussion

**Figure 1:**
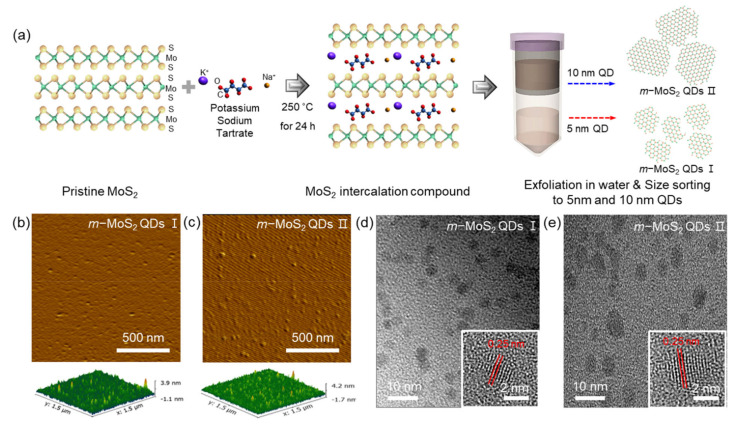
Schematic illustration and characterization of *m*-MoS_2_-QD I and *m*-MoS_2_-QD II (**a**) Fabrication steps of *m*-MoS_2_-QD I and *m*-MoS_2_-QD II. (**b**,**c**) AFM images. (**d**,**e**) HR-TEM images of *m*-MoS_2_-QD I and *m*-MoS_2_-QD II.

The overall synthetic route for the *m*-MoS_2_-QDs is shown in Figure 1a. The *m*-MoS_2_-QDs were fabricated by forming co-intercalation compounds using an alkali metal–organic compound (potassium sodium tartrate tetrahydrate salt) in bulk MoS_2_ powder. The exfoliation to the *m*-MoS_2_-QDs and extraction to different sizes of *m*-MoS_2_-QDs (~5 nm and ~10 nm) occurred through a filter centrifugation process (8000 and 10,000 NMWL, Amicon Ultra-15, Merck Millipore, Cork, Ireland). The synthesized *m*-MoS_2_-QDs were metallic phase and were well dispersed in water. Further details of the experimental method are described in Section 2. Using AFM and HR-TEM, the *m*-MoS_2_-QDs were successfully distinguished into two sizes. The thickness of the *m*-MoS_2_-QDs (Figure 1b,c and Figure S1) was distributed in the range of 2–3 nm, regardless of the lateral size, indicating that the *m*-MoS_2_-QDs are mono- or bilayers. The topological heights of all *m*-MoS_2_-QDs samples fall homogeneously under 4 nm. HR-TEM images of the *m*-MoS_2_-QDs are presented in Figure 1d,e and Figure S2, revealing the lateral sizes of ~5 nm (*m*-MoS_2_-QD I) and ~10 nm (*m*-MoS_2_-QD II), respectively. The size distribution of *m*-MoS_2_–QDs and *m*-MoS_2_–QDs showed ~83% in the range of 2 ~ 5 nm and ~79% in the range of 10 ~ 13 nm. All *m*-MoS_2_-QDs exhibited a highly crystalline structure with a lattice spacing of 0.25 nm, which is consistent with previous reports [12,25]. From the FFT, the *m*-MoS_2_-QDs have an octahedral crystalline structure, implying a metallic phase (Figure S3).

**Figure 2:**
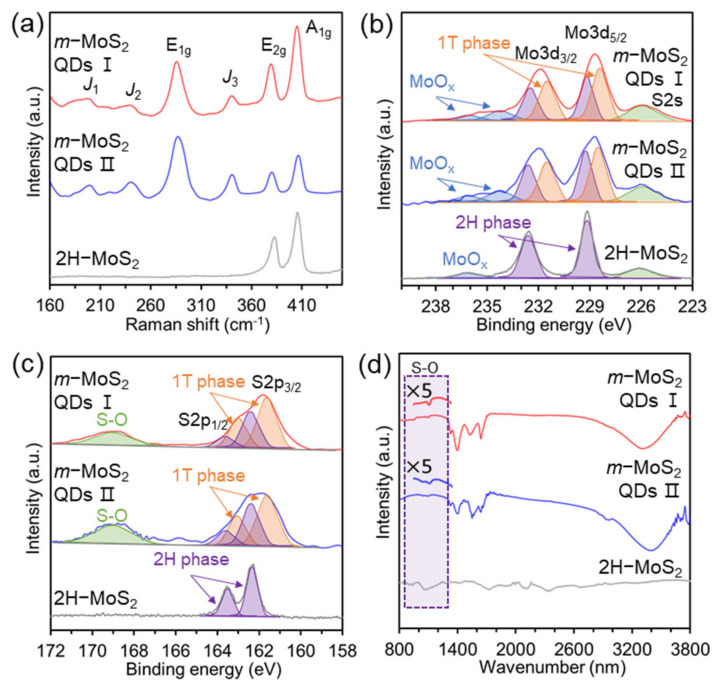
(**a**) Raman spectra of 2H-MoS_2_, *m*-MoS_2_-QD I, and *m*-MoS_2_-QD II. XPS spectra of molybdenum and sulfur elements Mo 3d of 2H-MoS_2_, *m*-MoS_2_-QD I, and *m*-MoS_2_-QD II (**b**). S 2p of 2H-MoS_2_, *m*-MoS_2_-QD I, and *m*-MoS_2_-QD II (**c**). (**d**) FT-IR spectra of 2H-MoS_2_, *m*-MoS_2_-QD I, and *m*-MoS_2_-QD II.

In Figure 2a, the specific vibrational modes of *m*-MoS_2_-QDs are presented as the *J*_1_*, J*_2_, and *J*_3_ bands at 146.5, 235, and 335.5 cm^−1^ in the Raman spectra, respectively [7,26,27], indicating the existence of metallic phase MoS_2_, while there are no peaks in 2H-MoS_2_. In addition, the characteristic modes of in-plane (E_2g_) and out-of-plane (A_1g_) vibrational modes in MX_2_ were observed regardless of the lateral size in all samples, and the relatively large-sized *m*-MoS_2_-QDs showed a blue shift owing to the strain induced by oxygen functional groups. These results indicate that the synthesized MoS_2_-QDs undergo a structural metallic phase transition, lattice vacancies, and an oxygen-rich phase through tartrate functionalization during strain-induced lattice deformation. Figure 2b,c show the XPS spectra for the Mo 3d, S 2s, and S 2p regions of the synthesized *m*-MoS_2_-QDs, which are in the metallic phase. The XPS spectra of the *m*-MoS_2_-QDs showed that the transition metal and chalcogen peaks are broadened with additional peaks in terms of lower binding energy (phase transition of MoS_2_ crystal from 2H to 1T) and higher binding energy (oxygen-related functional groups) compared to those of the semiconducting 2H phase MoS_2_ [28,29,30]. In addition, the Mo 3d and S 2p peaks of the *m*-MoS_2_-QDs are deconvoluted into seven peaks (226.1 eV for S 2s; 229.2 (2H) and 228.3 eV (1T) for Mo 3d_5/2_ (Mo4^+^); 232.6 (2H) and 231.7 eV (1T) for Mo 3d_3/2_ (Mo4^+^); 236.0 and 234.2 eV for MoO_x_) [31]. Interestingly, the Mo3d peaks related to the metallic phase (1T) were shifted ~0.9 eV lower than those of the 2H phase MoS_2_ in the Mo spectra. In addition, the different oxidation peaks of Mo and S in both samples were observed with peak broadening to higher binding energies, which was ascribed to decreased structural uniformity and increase in local strain compared to 2H-MoS_2._ The S 2p spectra of the *m*-MoS_2_-QDs revealed S2^−^ doublet peaks including 1T phase at 161. 7 and 163.1 eV and 2H phase at 162.3 and 163.7 eV, and the S-O peak was broadened at ~169.0 eV, which was ascribed to oxidation of sulfur [32]. These results are consistent with previous works [28,29,30,31,32]. Figure 2d shows the chemical bindings of the *m*-MoS_2_-QDs measured by FT-IR analysis. Both samples have the characteristic peaks of the S-O (from 1000~1200 cm^−1^), C-H band (2923 cm^−1^), C=O/COOH band (1720 cm^−1^), and -OH band (1379 cm^−1^) corresponding to the diverse oxidation states of Mo and S due to the presence of tartrate-related chemicals on *m*-MoS_2_-QDs [33]. These results indicate that functional groups ascribed to oxygen groups, doping, and vacancy defects are formed on the surface or at the edge of the *m*-MoS_2_-QDs. In addition, the dispersion stability is longer than one month because of the partial negative charge of the oxygen functional groups, as revealed by the zeta potential in Figure S4. From the zeta potential, *m*-MoS_2_-QD I has fewer oxygen functional groups than *m*-MoS_2_-QD II, resulting in important characteristic differences in the optical properties of these *m*-MoS_2_-QDs.

**Figure 3:**
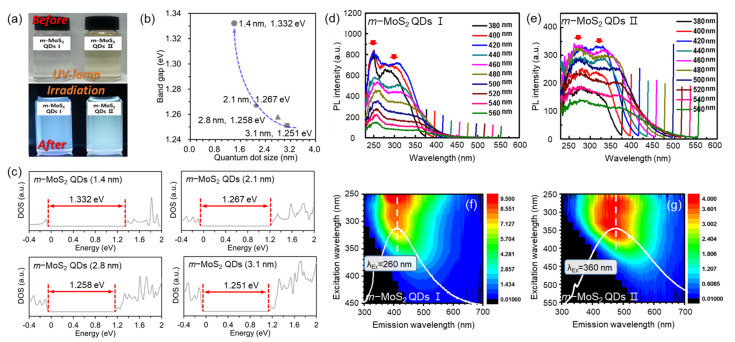
(**a**) Digital images of *m*-MoS_2_-QD I and *m*-MoS_2_-QD II. before (top) and after (down) UV illumination in water. (**b**) Bandgap of *m*-MoS_2_-QDs calculated by DFT. (**c**) Density of states of *m*-MoS_2_-QDs with different sizes. PLE spectra of *m*-MoS_2_-QD I (**d**) and *m*-MoS_2_-QD II (**e**) with varying excitation wavelength from 380 to 560 nm. 2D PLE spectra of PLE spectra of *m*-MoS_2_-QD I at 260 nm excitation (λ_Ex_) (**f**) and *m*-MoS_2_-QD II (**g**) at 360 nm excitation (λ_Ex_).

Figure 3a and Figure S5 show the digital images and UV–vis spectra of the synthesized *m*-MoS_2_-QDs, which were used to determine their optical properties. The absorption intensity of both *m*-MoS_2_-QD I (~5 nm) and *m*-MoS_2_-QD II (~10 nm) decreased because of the metallic property of the *m*-MoS_2_-QDs compared to that of 2H-MoS_2_-QDs. The absorption intensity of *m*-MoS_2_-QD I is larger than that of *m*-MoS_2_-QD II because of the notable difference in excitonic features caused by the quantum confinement effect. In addition, the emitting colors of *m*-MoS_2_-QD I and *m*-MoS_2_-QD II are blue and green, respectively, under the excitation of a 365 nm ultraviolet lamp. To understand the optical properties of the *m*-MoS_2_-QDs derived from different sizes, first-principles calculations within the DFT framework were performed on the representative structural model of 1T-MoS_2_. Figure 3b,c show the variation in the bandgap of the *m*-MoS_2_-QDs without defects with the QD size, where the bandgap increases as the size decreases owing to the quantum confinement effect.

In addition, the PLE spectra were measured at the maximum PL peak positions of both *m*-MoS_2_-QD I and *m*-MoS_2_-QD II, as shown in Figure 3d,e. The PLE spectra of the *m*-MoS_2_-QDs clearly showed two maximum peaks at every emission wavelength. The PLE spectra of the *m*-MoS_2_-QD I show a sharp peak at 240–260 nm (A-band), which originates from the stoichiometry of the m-MoS_2_-QDs as well as a broad shoulder at 290–375 nm (B-band) related to the oxygen defects. However, for the *m*-MoS_2_-QD II sample (A-band: 260–285 nm, B-band: 310–390 nm), it is difficult to find a sharp PLE peak due to the oxygen defects, and the maximum peaks were broadened and shifted to the longer wavelength region. The PLE of *m*-MoS_2_-QD II with higher oxygen functional groups was dominated by the B-band. These optical properties are consistent with the XPS, Raman, and zeta potential results. Interestingly, the difference of the two PLE peaks was 0.7–0.9 eV, which is similar to the valence band splitting in *m*-MoS_2_-QDs, and is due to the strong spin–valley coupling and the quantum confinement effect [12,18]. Figure 3f,g show the two-dimensional contour plots correlated with the excitation and emission wavelengths of *m*-MoS_2_-QD I and II. The maximum emission wavelength corresponded to two regions in the PLE spectra. As the excitation wavelength (λ_Ex_) increased from 250 nm to 550 nm, the emission peaks were red-shifted in *m*-MoS_2_-QD I and II.

The maximum emission peaks of *m*-MoS_2_-QD I were gradually red-shifted as the excitation wavelength increased from 250 to 450 nm. In contrast, the oscillation of the emission peak in *m*-MoS_2_-QD II was weakened, and the highest luminescence intensity shifted into a longer wavelength. These results indicate that the optical characteristics of the size-dependent *m*-MoS_2_-QDs originate from the quantum confinement effect and oxygen content.

**Figure 4:**
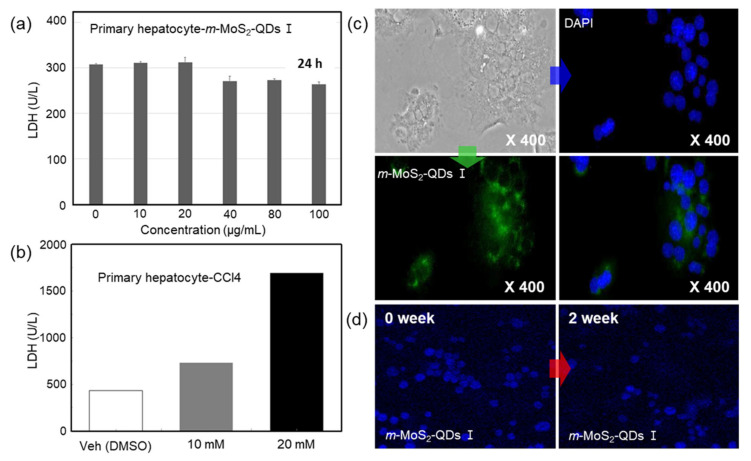
(**a**) Cytotoxicity for mouse primary hepatocytes. (**b**) Multiphoton images depending on *m*-MoS_2_-QD concentration. (**c**) Mouse primary hepatocyte images with *m*-MoS_2_-QD after 24 h. Phase contrast image (top left), nucleus stained with DAPI (top right), green fluorescence of *m*-MoS_2_-QD (bottom left), and overlay image of DAPI and green *m*-MoS_2_-QD (bottom right). (**d**) Imaging after 0 weeks (left) and after 2 weeks (right).

Finally, the cytotoxicity of the *m*-MoS_2_-QDs (5 nm) toward mouse-derived primary hepatocytes determined using an LDH detection assay is shown in Figure 4a. Interestingly, the *m*-MoS_2_-QDs did not impose significant toxicity on hepatocyte cells up to 100 μg mL^−1^ compared to that of the untreated control sample after 24 h of incubation. However, hepatocytes treated with CCl4 as a liver injury factor showed significantly higher LDH levels, as shown in Figure 4b. Figure 4c illustrates the fluorescent images of mouse-derived primary hepatocytes with highly concentrated *m*-MoS_2_-QDs. Although the nuclei of hepatocytes were stained with DAPI, the *m*-MoS_2_-QDs mainly stained the cell body rather than the nucleus and showed high green fluorescence after single-photon excitation in overlay images. Furthermore, the *m*-MoS_2_-QDs showed stability in the hepatocytes for two weeks, as shown in Figure 4d. This indicates that the *m*-MoS_2_-QDs are promising probes for bio-imaging and other biomedical applications.

## 4. Conclusions

This paper proposed a cost-effective and eco-friendly method for synthesizing *m*-MoS_2_-QDs with excellent dispersion in water and strong photoluminescence, without the use of any organic solvents or surfactants. The proposed method was easily scalable and applicable to various bio-related applications. From the structural and optical analyses, we confirmed that *m*-MoS_2_-QDs with different sizes were successfully fabricated, and we observed clear shifts of the characteristic peaks as the size of the *m*-MoS_2_-QDs varied. In addition, the fluorescence from the synthesized *m*-MoS_2_-QDs consisted of two overlapping bands originating from the stoichiometry of the MoS_2_-QDs (A-band) and oxygen defects (B-band). The emitting color was found to be controlled by changing the size and oxygen content of the *m*-MoS_2_-QDs because of the quantum confinement and surface states of the oxygen functional groups. Furthermore, the water-soluble *m*-MoS_2_-QDs can be used for imaging hepatocytes at near-infrared wavelengths and show excellent biocompatibility in the cells. Our studies suggest a general understanding of two-dimensional heteroatomic-structured QDs, providing potential applications for biosensors, disease diagnosis, and biological imaging. The research of water-soluble MoS_2_-QDs in memory and neuromorphic computing fields can lead to prospective utilization of brain–machine interfaces, robotic skin, and biosensor/optical sensor networks.

## Data Availability

Not applicable.

## Supplementary Materials

The following supporting information can be downloaded at: https://www.mdpi.com/article/10.3390/nano12101645/s1, Thickness distribution of *m*-MoS_2_-QDs by AFM (Figure S1); size distribution of *m*-MoS_2_-QDs by TEM (Figure S2); Fourier transform pattern of *m*-MoS_2_-QDs by TEM (Figure S3); Zeta potential of *m*-MoS_2_-QDs (Figure S4); UV–vis spectra of *m*-MoS_2_-QDs (Figure S5) (PDF).
