# Supplementary material for: Metallic Phase Transition Metal Dichalcogenide Quantum Dots as Promising Bio-Imaging Materials
Source: Nanomaterials (Basel). 2022 May 11;12(10):1645. doi: 10.3390/nano12101645 (PMC9144094; doi:10.3390/nano12101645)
Supplement: Supplementary file 1 [file nanomaterials-12-01645-s001.zip › nanomaterials-1633790-supplementary.pdf]

nanomaterials

Supplementary materials

Metallic phase transition metal dichalcogenide quantum dots as promising bio-imaging materials

Kwang Hyun Park [1], Jun Yong Yang [1], Sunggyeong Jung [1], Byoung Min Ko [1], Gian Song [1], Soon-Jik Hong [1], Nam Chul Kim [1,*], Dongju Lee [2,*] and Sung Ho Song [1,*]

[1] Division of Advanced Materials Engineering and Center for Advanced Powder Materials and Parts, Kongju National University, Cheonan 32588, Chungnam, Korea; recite14@gmail.com (K.H.P.); yajy2306@naver.com (J.Y.Y.); jsk71317@gmail.com (S.J.); qudals3920@gmail.com (B.M.K.); gasong@kongju.ac.kr (G.S.); hongsj@kongju.ac.kr (S.-J.H.)

[2] Department of Advanced Materials Engineering, Chungbuk National University, Chungdae-ro 1, Seowon-Gu, Cheongju 34057, Chungbuk, Korea

* Correspondence: nckim@kongju.ac.kr (N.C.K.); dongjulee@chungbuk.ac.kr (D.L.); shsong805@kongju.ac.kr (S.H.S.); Tel.: +82-0415219379 (S.H.S.)

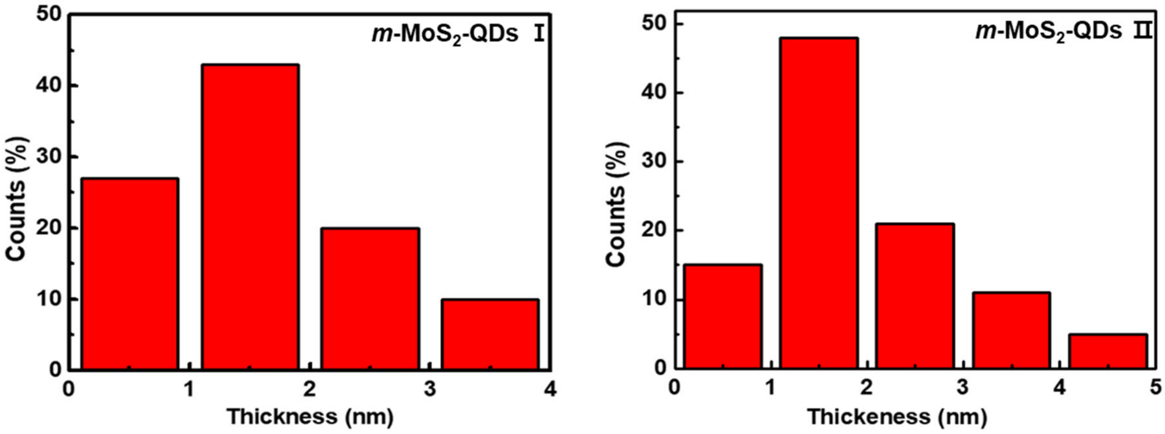

Figure S1. Thickness distribution of *m*-MoS_2-QDs by AFM.

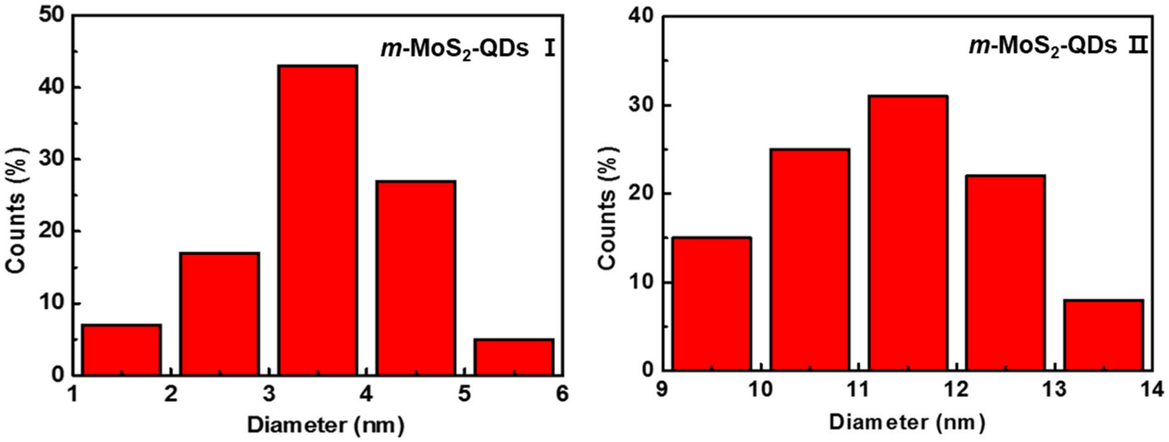

Figure S2 Size distribution of *m*-MoS_2-QDs by TEM

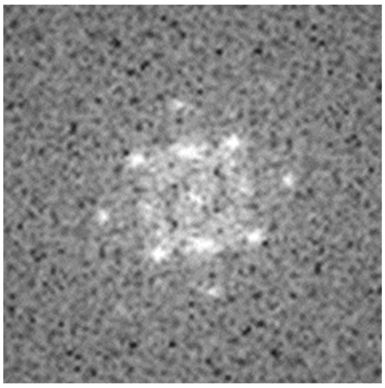

Figure S3. Fast Fourier transform pattern of *m*-MoS_2-QDs by TEM.

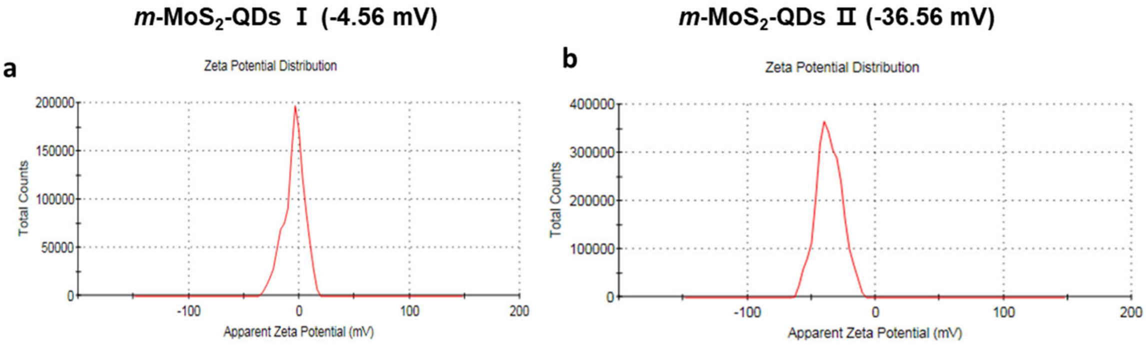

Figure S4. Zeta potential of *m*-MoS_2-QDs.

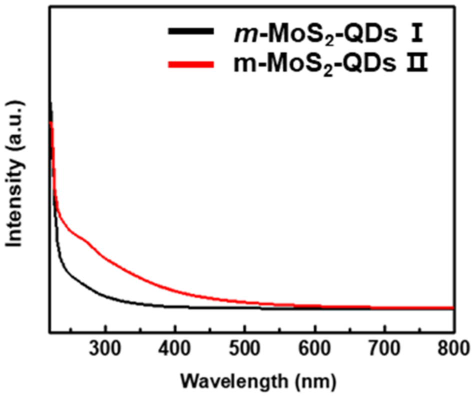

Figure S5. UV-vis spectra of *m*-MoS_2-QDs.
